# NT-proBNP and its correlation to left ventricular ejection fraction and heart failure – The DEMONSTRATE database

**DOI:** 10.1177/00045632251403397

**Published:** 2025-11-17

**Authors:** Morgan Lundgren, Peter Ridefelt, Maria K Svensson, Emil Hagström, Thomas Cars, Anders Larsson

**Affiliations:** 1 Department of Medical Sciences, Clinical Chemistry, 59592 Uppsala University, Uppsala, Sweden; 2 Department of Medical Sciences, Renal Medicine, 59592 Uppsala University, Uppsala, Sweden; 3 Uppsala Clinical Research Centre, Uppsala, Sweden; 4 Department of Medical Sciences, Cardiology, 83048 Uppsala University, Uppsala, Sweden; 5 Sence Research AB, Uppsala, Sweden

**Keywords:** Brain natriuretic peptide, left ventricular ejection fraction, heart failure, echocardiography, coronary disease

## Abstract

**Background:**

Measurement of N-terminal pro-B-type natriuretic peptide (NT-proBNP) and left ventricular ejection fraction (LVEF) are used in diagnosing heart failure (HF). The main aim was to explore the correlation between NT-proBNP and LVEF.

**Methods:**

Patient data for 14,962 patients were extracted from medical records and national registries and compiled in the Swedish DEMONSTRATE database. HF phenotype was categorized according to LVEF level: HF with reduced EF (≤40%, HFrEF); HF with mildly reduced EF (41–49%, HFmrEF); HF with preserved EF (≥50%, HFpEF). Spearman’s rank was employed for correlation analysis and ROC curves for discrimination and classification.

**Results:**

NT-proBNP correlated negatively with LVEF level (r = −0.40) and positively with age (r = 0.49), creatinine (r = 0.35), and cystatin C (r = 0.53). Individuals with an HF diagnosis were more likely to have higher NT-proBNP levels compared to those without. The association between NT-proBNP and LVEF remained statistically significant (*P* < .0001) also after adjusting for age and kidney function estimates (r = −0.20). NT-proBNP discriminated well between HFrEF (AUC = 0.80) and HFpEF (AUC = 0.78). In discriminating the presence of an HF diagnosis, NT-proBNP (AUC = 0.81) outperformed LVEF (AUC = 0.75). However, on an individual level the correlation between LVEF and NT-proBNP was modest.

**Conclusions:**

NT-proBNP levels increase when LVEF deteriorates but with large inter-individual differences. Further research is needed, but these findings show potential in optimizing the use of LVEF with the aid of sequential analysis of NT-proBNP as a complementary diagnostic and prognostic tool to enhance assessment of cardiac function.

## Introduction

Heart failure (HF) is one of the leading causes of hospitalization worldwide and has a significant mortality rate.^1,2^ At presentation, the symptoms and signs are often relatively unspecific.^1^ The criteria consist of having symptoms of HF that may be accompanied by clinical signs and having a reduced left ventricular ejection fraction (LVEF), or, if LVEF is preserved, objective evidence of cardiac abnormalities consistent with left ventricular dysfunction, such as raised natriuretic peptides.^1^ Due to the non-specific symptoms of HF, it is important to have cost-effective screening tools with high sensitivity to be able to efficiently rule out HF.^3,4^

According to European Society of Cardiology (ESC) guidelines, when chronic HF is suspected, it is recommended to analyse the cardiac biomarker N-terminal pro-B-type natriuretic peptide (NT-proBNP).^1^ NT-proBNP originates from B-type natriuretic peptide (BNP), which is synthesized by cardiomyocytes during elevated intracardiac pressure.^5,6^ If NT-proBNP is < 125 ng/L, chronic HF is unlikely and other diagnoses should be considered, but if NT-proBNP is ≥ 125 ng/L, it is recommended to perform an echocardiography. The value for classifying acute HF as unlikely is NT-proBNP <300 ng/L. Using echocardiography, the HF phenotype is based on LVEF, where LVEF ≤40% is defined as ‘HF with reduced ejection fraction’ (HFrEF), 41–49% as ‘HF with mildly reduced ejection fraction’ (HFmrEF), and ≥50% as ‘HF with preserved ejection fraction’ (HFpEF).^1^ Furthermore, LVEF ≤30% is regarded as severely reduced, 50–55% as normal in the lower range, and ≥55% as normal.^1,7^

Previous smaller studies have observed a negative correlation ranging from −0.333 to −0.721 between NT-proBNP and LVEF,^8–11^ which included one study with 348 participants with dyspnoea of cardiac origin focussing mostly on correlation to NYHA (New York Heart Association) class^9^ and one study with 79 patients with unstable angina with preserved LVEF and diabetes mellitus.^11^ One study with 37 heart failure patients with different co-morbidities noted a mean NT-proBNP of 17,303 (±12,174) ng/L when LVEF was <40%, 8162 (±9434) ng/L when LVEF was 40–50%, and 2272 (±3084) ng/L when LVEF was >50%.^10^ Another study with 100 patients >60 years with dyspnoea due to either cardiac or non-cardiac reasons displayed a mean NT-proBNP of 2764 ng/L when LVEF was <30%, 2092 ng/L when LVEF was 30–39%, 1359 ng/L when LVEF was 40–49%, and 892 ng/L when LVEF was ≥50%.^8^ Having HF management strategies which rely more on sequential NT-proBNP testing rather than echocardiographies to rule out HF would increase the cost-effectiveness of managing patients with suspected or diagnosed HF.^4^

Elevated concentrations of NT-proBNP can also be noted with increasing age, as well as in patients with coronary heart disease (CHD) or decreased kidney function.^12–14^ For instance, patients with dyspnoea of non-cardiac origin had lower NT-proBNP concentrations (mean = 309 ng/L) than patients with dyspnoea of cardiac origin (mean = 2345 ng/L) in one study.^8^ In that study, patients who expired had higher mean NT-proBNP concentrations, possibly hinting at its usefulness as a prognostic tool in this regard.^8^ There is also a sex difference, with females generally having higher concentrations of NT-proBNP than males.^13^ Apart from these factors, differences in NT-proBNP levels can be observed due to methodological causes, with methods from different manufacturers measuring at different levels due to bias and the lack of a universal reference standard.^15,16^ Due to these reasons and because HF guidelines usually do not take age or method into consideration, evaluation of NT-proBNP results in a clinical setting might prove complex.

The main aim of this study was to explore the correlation between laboratory measurements of NT-proBNP and echocardiographic estimations of LVEF, and the association between NT-proBNP levels and risk of hospitalization and mortality was evaluated. Previous studies are smaller, usually focussing on dyspnoea, NYHA classes and sometimes on other patient groups, and present mean values rather than correlations between NT-proBNP and LVEF classifications. Additionally, to strengthen the validity of the study cohort, the influence of sex, age, and biomarkers for kidney function (i.e. cystatin C, creatinine, and cystatin C- and creatinine-based estimated glomerular filtration rate [eGFR]) on NT-proBNP concentrations was explored.

## Materials and methods

This population-based retrospective observational cohort study utilizes data from the DEMONSTRATE database, which includes data from Swedish electronic medical records and national registries, such as the national patient registry.^17^ The DEMONSTRATE database was originally created for studying hyperkalaemia in patients regarding clinical events, treatment pathways, and usage of healthcare resources. Thus, entry requirements for patients in the DEMONSTRATE database were to have at least one potassium (K^+^) value, regardless of concentration, and a diagnosis of HF, hyperkalaemia, chronic kidney disease, diabetes, or hypertension registered in their electronic medical record. Exclusion criteria were being younger than 18 years old and not having a valid Swedish personal identification number. The study setting is Uppsala, Sweden, and the study period is 1^st^ of January 2011 to 31^st^ of December 2022.

According to the recommendations of investigating HF, NT-proBNP values above the cut-off are followed by an echocardiography. Due to the structure of the healthcare system, sometimes there is a delay or gap between the NT-proBNP measurement and the echocardiography. In this study, this gap was allowed to be at maximum 60 days. This time limit was chosen to avoid excluding too many potential study participants and not having too long between NT-proBNP and LVEF results. Thus, the target patient population was every patient regardless of diagnosis in the DEMONSTRATE database that fulfilled the criteria of having results of both NT-proBNP and LVEF within the allotted timeframe. These criteria were fulfilled for 14 952 patients.

LVEF was estimated visually or with the biplane Simpson’s method in the majority of the cases. If a patient had multiple LVEF results during the study period, only the first instance of an LVEF result with an NT-proBNP value within 60 days prior to the LVEF result was included. If multiple NT-proBNP values existed within the 60-day period, the NT-proBNP value closest to the LVEF result was chosen. Due to the study design, some of the patients included already had an established diagnosis of HF and/or echocardiographies performed prior to the start of the study period. If not mentioned otherwise, these patients with already established HF diagnoses or prior echocardiographies were included in the analyses as any other participant.

NT-proBNP was measured immunologically with either the Roche Cobas e601/801 (*n* = 10,111) or Abbott Architect i2000SR (*n* = 4851) method. The analytical range is 5–35,000 ng/L for Roche Cobas and 8–35,000 ng/L for Abbott Architect, with samples >35,000 being diluted up to 70,000 ng/L due to a local routine of the laboratory at Uppsala University Hospital. In a Swedish EQA study containing data from Uppsala University Hospital, Abbott methods tended to yield results 8% higher at lower levels (median = 141 ng/L, range = 27–777 ng/L) and 6% higher at higher levels (median = 667 ng/L, range = 181–10,740 ng/L) compared to Roche methods. Additionally, imprecision for Abbott methods was 6% at lower levels and 5% at higher levels. The corresponding numbers for Roche methods were 6% and 4%, respectively.^15^

A numerical LVEF value was extracted from echocardiography reports. If LVEF was reported as an interval or as several different values, the mean value was used. If LVEF was reported as ‘greater than or equal to’ or ‘less than or equal to’, the value was interpreted as ‘equal to’. If LVEF was reported as ‘greater than’ or ‘less than’, the value closest to the reported value was used (i.e. <30% was interpreted as 29%, and >30% as 31%), with one exception for the most common result, LVEF >55%, which was always interpreted as 55%.

Plasma measurements of cystatin C with an immunoturbidimetric method on Cobas and creatinine with an enzymatic method on Cobas were also extracted and included when present and within 60 days prior to the LVEF examination. Creatinine-based eGFR in mL/min/1.73 m^2^ body surface area (BSA) was calculated from the DEMONSTRATE-extracted medical record data on creatinine, age, and sex according to the revised Lund-Malmö (LM rev) equation,^18^ while cystatin C-based eGFR was calculated according to the CAPA (Caucasian, Asian, Paediatric and Adult cohorts) equation.^19^ Reference intervals were collected from Uppsala University Hospital, Sweden, where all analyses were performed (https://labhandbok.se/). Due to fewer results for cystatin C compared to creatinine, calculations on cystatin C-based eGFR have been omitted in some subgroups.

Other variables collected were age, sex, hospitalization due to HF or any cause, and death due to CHD or any cause. Data on the presence of HF was defined as having a diagnosis code starting with I50, based on the 10^th^ revision of the International Classification of Diseases (ICD-10). For diagnosis of CHD, ICD-10 codes I20–I25 were used.

Box plots with whiskers were used to show the variability of NT-proBNP values when categorizing LVEF values. The boxes indicate median values and values for the 1^st^ and 3^rd^ quartiles. The whiskers indicate maximum and minimum values without outliers, where outliers were defined as values outside of 1.5 times the interquartile range (IQR).

Spearman’s rank correlation was used to measure the strength and direction (i.e. positive or negative) of association between NT-proBNP and LVEF, age, cystatin C above reference interval, creatinine above reference interval, and eGFR, respectively. Partial correlations were used to calculate the association between NT-proBNP and LVEF when adjusting for age ≥60 years; cystatin C >1.10 mg/L for ages 18–49, >1.20 mg/L for ages 50–59, >1.30 mg/L for ages 60–69, and >1.40 mg/L for ages ≥70; creatinine >90 micromol/L for females and >105 micromol/L for males, and eGFR <60 (classified as ‘mild to moderate loss of kidney function’ according to Kidney Disease Improving Global Outcomes, KDIGO, guidelines^20^). Differences between two groups were assessed by using an independent t-test or a Mann–Whitney U-test.

Receiver operation characteristic (ROC) curves with corresponding area under the curve (AUC) and 95% confidence intervals (CIs) were used in order to evaluate NT-proBNP when discriminating between LVEF categories (HFrEF ≤40%, HFmrEF 41–49%, or HFpEF ≥50% according to ESC guidelines^1^), presence of HF diagnosis, death by CHD, and whether a patient received inpatient or outpatient care due to HF. Corresponding ROC calculations were performed to evaluate LVEF when discriminating between the presence of an HF diagnosis, death by CHD, and whether a patient received inpatient or outpatient care due to HF. The Youden index J was used to determine optimal thresholds for classification.

Kaplan–Meier analysis was used to analyse time-to-event data, in this case time to HF diagnosis for patients without prior HF diagnosis based on NT-proBNP and LVEF.

Statistical analyses were performed by using Excel 365 (Microsoft Corp., Seattle, WA, USA) and MedCalc for Windows, version 20.217 (MedCalc Software, Ostend, Belgium). Statistical significance was defined as a *P*-value <.0001 since no correction was made for multiple testing.

The study was conducted in accordance with the Declaration of Helsinki (*Br Med. J* 1964; **ii:** 177) and approved by the Swedish Ethical Review Authority (Dnr 2022-01051-01, 2024-04959-02).

## Results

### Biochemical and clinical characteristics of the study population

**Figure 1.**
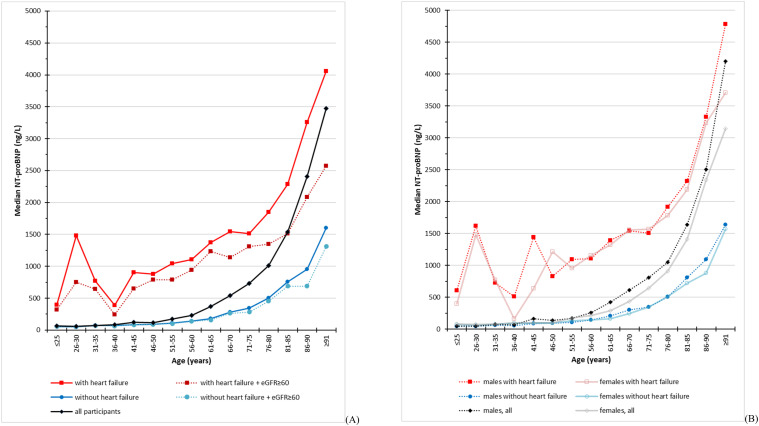
Age (*x*-axis) and median NT-proBNP (*y*-axis) in patients sorted on presence of heart failure diagnosis and (a) kidney function or (b) sex.

The biochemical and clinical characteristics of the 14,962 patients included are summarized in Table 1. Differences in median NT-proBNP concentrations between Abbott Architect (median = 610 ng/L, IQR = 166–2310 ng/L) and Roche Cobas (median = 661 ng/L, IQR = 159–2237 ng/L) were not statistically significant (*P*-value = .8236). Elevation of NT-proBNP was noted with increasing age, particularly for ages ≥75 years, regardless of sex. More males (44%) than females (40%) had a diagnosis of HF. Although not statistically significant (*P*-value = .0819), the unpaired t-test displayed a sex difference, with females having higher mean NT-proBNP concentrations than males by 189 ng/L (95% CI = −24–403 ng/L). On the other hand, males in general exhibited slightly higher median NT-proBNP levels compared to females. Higher NT-proBNP was observed in patients with a diagnosis of HF compared to patients without a diagnosis regardless of age and sex. Having eGFR <60 mL/min/1.73 m^2^ contributed to higher NT-proBNP, especially in age groups ≥75 years (Figure 1(a) and (b), Table S1).

**Table 1.** Biochemical and clinical characteristics of the patients included in this study.

Outcome characteristics	Male (*n* = 8059)	Female (*n* = 6903)	All (*n* = 14,962)
	*n*	*n*	*n*
Heart failure diagnosis total	3583	2779	6362
Heart failure diagnosis prior to NT-proBNP	1743	1308	3051
Heart failure diagnosis prior to LVEF^a^	2267	1774	4041
Heart failure-caused inpatient care	2447	2048	4495
All-cause inpatient care	5202	4468	9670
Coronary heart disease death	960	818	1778
All-cause death	2405	1932	4337
Creatinine results	7834	6,632	14,466
Cystatin C results	1836	1223	3059

Clinical and biochemical characteristics	Male		Female		All	
	Median	IQR	Median	IQR	Median	IQR
Age, years	71	61–78	74	61–81	72	61–80
NT-proBNP, ng/L	679	167–2194	598	157–2341	643	162–2260
LVEF^a^, %	51	45–55	55	51–55	55	48–55
Days between NT-proBNP–LVEF^a^, days	5	1–23	5	1–26	5	1–24
Creatinine, micromol/L	89	77–108	71	61–89	82	68–101
Cystatin C, mg/L	1.3	1.0–1.8	1.3	1.0–1.8	1.3	1.0–1.8
Creatinine-based eGFR, mL/min/1.73 m^2^ BSA	66	52–79	64	50–80	66	51–79
Cystatin C-based eGFR, mL/min/1.73 m^2^ BSA	53	35–74	54	34–79	53	35–74

^a^LVEF = left ventricular ejection fraction.

Males had more test results available (i.e. creatinine, cystatin C, and eGFR) compared to females. Median creatinine concentration was higher for males (89 micromol/L) compared to females (71 micromol/L), while both sexes had a similar median creatinine-based eGFR of 66 and 64 mL/min/1.73 m^2^, respectively. Cystatin C-based eGFR yielded lower results compared to creatinine-based eGFR. In patients with both creatinine-based and cystatin C-based eGFR, the results were in agreement 72% of the time when deciding whether eGFR is ≥ 60 or <60 mL/min/1.73 m^2^. The correlation between the two eGFR equations was R^2^ = 0.555. Other clinical characteristics such as age, inpatient care due to HF, and death by CHD were similar between the sexes. As for the subset with available cystatin C results, there was a significant difference (*P*-value <.0001) in median NT-proBNP between having a cystatin C result (median = 1100 ng/L, IQR = 277–3999 ng/L) and not having one (median = 555 ng/L, IQR = 144–1960 ng/L). The occurrence of heart failure diagnosis was 8% more common in the mentioned subset, and similarly, the occurrence of death was 9% more common. Age, LVEF, creatinine, and inpatient care were similar regardless of having a cystatin C result or not.

### Assessment of NT-proBNP and left ventricular ejection fraction

**Figure 2.**
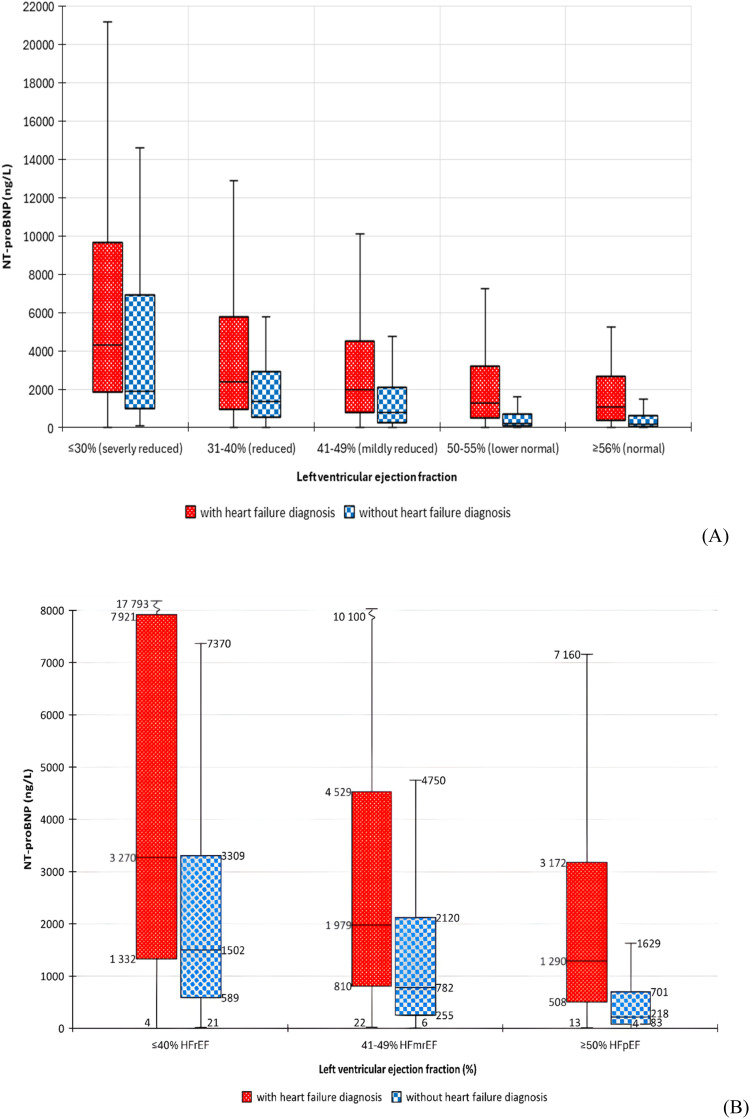
Correlation between left ventricular ejection fraction (*x*-axis) and NT-proBNP (*y*-axis) in patients with (boxes with dots) and without (boxes with squares) a diagnosis of heart failure. The box plots indicate values for the 1^st^ quartile, median, and 3^rd^ quartile, while the whiskers indicate maximum and minimum values without outliers. (a) Left ventricular ejection fraction according to physiological ranges. (b) Left ventricular ejection fraction according to heart failure phenotype with corresponding NT-proBNP concentration added to the boxes and whiskers.

The median LVEF for females was 55% and 51% for males (Table 1). When the LVEF was categorized according to different clinical stages, NT-proBNP was substantially higher in the patients with lower LVEF (Figure 2(a) and (b)). The patterns were consistent among patients with or without an HF diagnosis, although the absolute values for NT-proBNP were noticeably higher in HF patients. NT-proBNP is negatively correlated with LVEF (r = −0.404), and the correlation between NT-proBNP and LVEF weakened when adjusting for age (r = −0.315), creatinine (r = −0.284), creatinine-based eGFR (r = −0.294), cystatin C (r = −0.222), and cystatin C-based eGFR (r = −0.225) (Table S2). Furthermore, NT-proBNP was positively correlated with cystatin C, age, and creatinine and negatively correlated with eGFRs. The strongest correlations for NT-proBNP were with cystatin C-based eGFR (r = −0.545), cystatin C (r = 0.534), and creatinine-based eGFR (r = −0.515). Based on point estimates, all observed r-values, except for non-adjusted LVEF, were higher for females compared to males.

### Classification and discrimination values for NT-proBNP and left ventricular ejection fraction

Classification and discrimination values for NT-proBNP and LVEF are listed in Table 2. NT-proBNP performed well in discriminating between LVEF ≤40% (HFrEF) (AUC = 0.800) and LVEF ≥50% (HFpEF) (AUC = 0.781) and the presence of an HF diagnosis (AUC = 0.814). Based on point estimates, the observed AUC and threshold values for discriminating between LVEF categories with NT-proBNP were higher for females than males. On the other hand, it was noted that males had lower thresholds and larger AUCs compared to females when using LVEF to discriminate the presence of an HF diagnosis, death by CHD, and hospitalization due to HF. NT-proBNP (AUC = 0.814) outperformed LVEF (AUC = 0.751) in discriminating the presence of an HF diagnosis. The AUCs were lower (AUC = <0.700) in discriminating LVEF 41–49% with NT-proBNP and death by CHD and hospitalization due to HF regardless of using NT-proBNP or LVEF.

**Table 2.** Classification and discrimination values for NT-proBNP and left ventricular ejection fraction.

NT-proBNP	Threshold (ng/L)	Classification		AUC	95% CI	Youden index J	*P*-value	Current cases (n)	Total (n)
		Sensitivity (%)	Specificity (%)						
LVEF^a^ ≤40% – All	>1147	76	69	0.800	0.794–0.807	0.458	<0.0001	2,514	14,962
Male	>984	78	67	0.798	0.789–0.807	0.453	<0.0001	1726	8059
Female	>1585	76	74	0.820	0.811–0.829	0.495	<0.0001	788	6903
All ≥60 years	>1362	76	67	0.779	0.772–0.787	0.423	<0.0001	2,123	11,488
Male ≥60 years	>1362	74	69	0.779	0.768–0.789	0.423	<0.0001	1440	6178
Female ≥60 years	>1585	78	68	0.800	0.789–0.810	0.460	<0.0001	683	5310
LVEF^a^ 41–49% – All	>640	72	52	0.646	0.638–0.653	0.238	<0.0001	1,415	14,962
Male	>485	75	45	0.619	0.609–0.630	0.201	<0.0001	882	8059
Female	>755	74	57	0.686	0.675–0.697	0.303	<0.0001	533	6903
All ≥60 years	>750	74	47	0.626	0.617–0.634	0.211	<0.0001	1,196	11,488
Male ≥60 years	>671	73	44	0.600	0.587–0.612	0.173	<0.0001	735	6178
Female ≥60 years	>1310	68	60	0.666	0.653–0.679	0.285	<0.0001	461	5310
LVEF^a^ ≥50% – All	≤750	64	79	0.781	0.774–0.788	0.431	<0.0001	11,033	14,962
Male	≤708	65	78	0.782	0.773–0.791	0.429	<0.0001	5451	8059
Female	≤1189	72	74	0.795	0.785–0.804	0.455	<0.0001	5582	6903
All ≥60 years	≤1081	65	75	0.762	0.754–0.770	0.396	<0.0001	8,169	11,488
Male ≥60 years	≤1092	67	73	0.764	0.754–0.775	0.398	<0.0001	4003	6178
Female ≥60 years	≤1380	68	74	0.777	0.765–0.788	0.424	<0.0001	4166	5310
Heart failure diagnosis – All	>635	78	70	0.814	0.808–0.820	0.483	<0.0001	6,362	14,962
Male	>703	76	72	0.815	0.806–0.823	0.484	<0.0001	3583	8059
Female	>585	79	69	0.814	0.805–0.823	0.486	<0.0001	2779	6903
All ≥60 years	>836	74	68	0.781	0.773–0.788	0.445	<0.0001	5,715	11,488
Male ≥60 years	>838	75	69	0.786	0.775–0.796	0.474	<0.0001	3143	6178
Female ≥60 years	>731	76	65	0.776	0.765–0.787	0.419	<0.0001	2572	5310
Coronary heart disease death – All	>1851	63	56	0.615	0.601–0.630	0.189	<0.0001	1,778	14,962
Male	>1903	60	57	0.602	0.582–0.622	0.173	<0.0001	960	8059
Female	>1497	71	51	0.629	0.608–0.651	0.217	<0.0001	818	6903
Heart failure inpatient care – All	>2318	50	66	0.597	0.585–0.609	0.160	<0.0001	4,495	14,962
Male	>2553	46	71	0.603	0.586–0.619	0.167	<0.0001	2447	8059
Female	>2200	52	63	0.588	0.569–0.606	0.156	<0.0001	2048	6903

Left ventricular ejection fraction	Threshold (%)	Sensitivity (%)	Specificity (%)	AUC	95% CI	Youden index J	*P*-value	Current cases (n)	Total (n)
Heart failure diagnosis – All	≤50	54	88	0.751	0.744–0.758	0.421	<0.0001	6,362	14,962
Male	≤48	58	89	0.784	0.775–0.793	0.476	<0.0001	3583	8059
Female	≤50	43	92	0.710	0.699–0.720	0.348	<0.0001	2779	6903
Coronary heart disease death – All	≤50	54	65	0.626	0.611–0.640	0.186	<0.0001	1,778	14,962
Male	≤48	57	65	0.649	0.630–0.668	0.222	<0.0001	960	8059
Female	≤50	43	73	0.604	0.582–0.626	0.163	<0.0001	818	6903
Heart failure inpatient care – All	≤24	11	92	0.510	0.498–0.523	0.036	0.1809	4,495	14,962
Male	≤23	15	91	0.521	0.505–0.538	0.053	<0.0001	2447	8059
Female	≤53	61	43	0.517	0.498–0.536	0.043	0.1582	2048	6903

^a^Left ventricular ejection fraction.

### Time to heart failure diagnosis based on NT-proBNP and left ventricular ejection fraction

Time to HF diagnosis for patients without an existing HF diagnosis prior to study inclusion was analysed based on cut-off values for NT-proBNP and LVEF classifications (Table S3).

In general, earlier HF diagnosis was observed in higher NT-proBNP and lower LVEF values. Time to HF diagnosis was the shortest for LVEF ≤40% (median 44 days), followed by NT-proBNP above the rule-in criteria ≥300 ng/L (median 49 days) or ≥125 ng/L (median 62 days), and LVEF 41–49% (median 131 days). Except in cases with NT-proBNP ≥300 ng/L, LVEF ≤40%, or LVEF ≥50%, males had shorter median times to an HF diagnosis compared to females when looking at point estimates.

## Discussion

To our best knowledge, this is the first study in which NT-proBNP and LVEF measurements have been extracted from electronic health records and used to assess the correlation between NT-proBNP and LVEF, risk of hospitalization, and death in patients with and without a diagnosis of HF, along with also assessing the influence of sex, age, creatinine, cystatin C, and eGFR on NT-proBNP concentrations.

When comparing the cohort of patients according to phenotypical stages of LVEF, it was evident that a lower LVEF was accompanied by a higher NT-proBNP. However, there was a significant variability of NT-proBNP levels within each LVEF group. This is in line with a previous study^21^ and previous findings for NT-proBNP levels, where clinically stable HF patients displayed considerable within-hour (7%) and within-week (21%) intra-individual variability.^22^ Similar within-week intra-individual variability (24%) has been displayed among healthy subjects, while inter-individual variability (8%) is smaller than intra-individual variability.^23^ Consequently, it is less likely that a certain NT-proBNP can accurately predict LVEF for an individual than on a group level. Nevertheless, it is still possible that sequential analysis of NT-proBNP could predict corresponding changes in LVEF, thus being a highly useable biomarker at the individual level when the patient is compared to itself and its own previous measurements of NT-proBNP. For instance, one study demonstrated the value of sequential NT-proBNP analysis in HF patients where any 2.7-factor increase and any ≤60% decrease were accompanied with higher mortality risk.^24^

As expected, declining LVEF and kidney function and increasing age were positively correlated to NT-proBNP. These correlations are in line with previous findings.^8–14,21^ Since the other studies utilize different patient cohorts and mean NT-proBNP concentrations instead of medians for each LVEF classification, it makes comparison between studies difficult. It was noted that cystatin C seemed to be requested more often in a sicker population, possibly due to needing more accurate estimates on kidney function in critically ill patients with a lower muscle mass and a higher degree of inflammation. Age, cystatin C, creatinine, and eGFRs had, based on point estimates, stronger correlations to NT-proBNP in females than males, while LVEF was more strongly correlated to NT-proBNP in males. Since NT-proBNP concentrations in females seem to be more affected by age and lower kidney function, this could possibly be one of the reasons why females in general have higher NT-proBNP concentrations than males. The t-test, although not statistically significant, indicated females having higher mean NT-proBNP concentrations compared to males, while males in general had higher median NT-proBNP concentrations. More studies are needed to elucidate the influence of age and sex, especially in the population of ≥90-year-olds.

Males were more often assigned an HF diagnosis and had shorter times to diagnosis when NT-proBNP was elevated or LVEF was mildly reduced compared to females. This is also in line with previous findings and could possibly be explained by factors such as females having a tendency to present HF-related symptoms at an older age while simultaneously having several co-morbidities.^25^ NT-proBNP showed the potential in discriminating between LVEF ≤40% and LVEF ≥50%, with less precision for LVEF 41–49%. This could be due to HFmrEF (EF 41–49%) being introduced in the 2016 ESC guidelines^26^ and thus not existing during the first 5 years of the study period. Another possible explanation is that LVEF estimation on echocardiographies is semi-subjective, often done visually and reported in ranges sometimes spanning from 35% to 50%. The NT-proBNP thresholds were similar, >635–640 ng/L, for categorizing LVEF 41–49% and future HF diagnosis, indicating HF diagnoses being set when LVEF is reduced. This threshold is well above the rule in criteria for HF being likely at ≥125 ng/L and ≥300 ng/L. NT-proBNP performed better than LVEF in discriminating the existence of an HF diagnosis, possibly due to echocardiographies being conducted for various indications with LVEF sometimes being temporarily reduced due to conditions such as myocardial infarction. In contrast, NT-proBNP is primarily used for diagnosing HF. NT-proBNP and LVEF seem to convey similar information regarding discriminating the presence of inpatient care and death, which is in line with previous studies where one study noted that a worse NYHA functional class predicted mortality better than NT-proBNP and LVEF.^21,24^

ESC guidelines mention that NT-proBNP has high sensitivity and that normal levels in patients with suspected acute HF make the diagnosis unlikely, but also that 20% of patients with proven HFpEF have NT-proBNP concentrations below the decision limit.^1,26^ Because NT-proBNP increases with age, important decision limits might need to be stratified by age. In the case of HF, it would be preferred to detect optimal decision limits with increased sensitivity for younger patients with lower concentrations while keeping specificity for older patients with higher concentrations.^27^ The results from this study suggest that a diagnosis of acute or chronic HF could be established at NT-proBNP >635 ng/L regardless of age or sex with 78% sensitivity and 70% specificity. In this case, sensitivity is too low compared to the age-specific ESC limits for ruling in acute HF (>450 ng/L for <50 years, >900 ng/L for ages >50–75 years, and 1800 ng/L for >75 years), which have around 90% sensitivity and 84% specificity.^27^ Another study using identical age-specific cut-offs for acute HF yielded somewhat lower values, with sensitivity around 76–86% and specificity around 75–94% depending on age group.^28^ In comparison, one study presented the following sensitivity and specificity for age-specific decision limits in suspected chronic HF: 50 ng/L for <50 years, 99% and 57%; 75 ng/L for 50–75 years, 96% and 51%; and 250 ng/L for >75 years, 88% and 54%.^29^ Due to a limitation in the DEMONSTRATE database, it was not possible to discern acute from chronic HF, which could possibly explain the discrepancy between our data and the other studies.

The strength of this study was that it included almost 15 000 patients compared to previous similar studies with not more than a few hundred participants. One limitation is that LVEF is typically not reported as a numeric value but rather in ranges or less than a certain value, which introduces a degree of uncertainty to the ‘true’ value. Thus, there might have been other possibilities to handle LVEF data than, for instance, using mean values or nearest integers. To mitigate this problem, LVEF classifications have been used where applicable. Another limitation is that all included patients were examined with echocardiography, which could have introduced a selection bias for patients with potential other cardiovascular diseases besides HF. During the study period, the recommendation of echocardiography as first-line imaging in HF management was unchanged by ESC guidelines.^1,26,30,31^ However, echocardiography is also indicated for other conditions, such as valvular heart disease, acute coronary syndrome, and chronic coronary syndrome.^32^ This could potentially have selected a study cohort with lower LVEF and/or higher NT-proBNP results compared to the general population. Treatment was not considered; thus, patients might display better LVEF and lower NT-proBNP compared to at the time of HF diagnosis. Also, only the first LVEF result with an NT-proBNP value within 60 days was included in the study without information about the current clinical situation of the individual patient. Thus, the patient might be suffering from undiagnosed HF or, in contrast, be optimally treated for HF at the time of sampling, which in turn could affect the results of LVEF and NT-proBNP. Unfortunately, due to this being a retrospective study, information about body mass index (BMI) or diagnosis of atrial fibrillation was not available, which would have been interesting to investigate given their relationship to HF and them being known confounders.

In conclusion, this large real-world cohort study shows that NT-proBNP levels increase when LVEF deteriorates, although there are substantial inter-individual differences. The negative correlation between NT-proBNP and LVEF remains statistically significant when adjusting for age and markers of kidney function. NT-proBNP has the ability to discriminate between HFrEF and HFpEF, and it outperforms LVEF in discriminating existence of HF diagnosis relatively well. An enhanced understanding of the correlation between NT-proBNP and LVEF can improve assessment of cardiac function and risk of CHD. Although further studies are needed, this study holds the potential in optimizing the use of LVEF with the aid of sequential analysis of NT-proBNP as a complementary diagnostic and prognostic tool to enhance assessment of cardiac function.

## Supplemental Material

Supplemental Material - NT-proBNP and its correlation to left ventricular ejection fraction and heart failure – The DEMONSTRATE database Supplemental Material for NT-proBNP and its correlation to left ventricular ejection fraction and heart failure – The DEMONSTRATE database by Morgan Lundgren, Peter Ridefelt, Maria K Svensson, Emil Hagström, Thomas Cars, and Anders Larsson in Annals of Clinical Biochemistry
