# Supplementary material for: NT-proBNP and its correlation to left ventricular ejection fraction and heart failure – The DEMONSTRATE database
Source: Ann Clin Biochem. 2025 Nov 17;63(4):308–18. doi: 10.1177/00045632251403397 (PMC13332166; doi:10.1177/00045632251403397)
Supplement: Supplemental Material - NT-proBNP and its correlation to left ventricular ejection fraction and heart failure – The DEMONSTRATE database [file sj-pdf-1-acb-10.1177_00045632251403397.pdf]

Supplemental Data

Supplemental Data Table S1. NT-proBNP values at different ages non-grouped and grouped by presence of heart failure diagnosis, kidney function, and sex.

**Patients regardless of heart failure and kidney function**				
Age (years)	NT-proBNP			N
	Q1 (ng/L)	Median (ng/L)	Q3 (ng/L)	
≤25	28	65	199	280
26–30	29	58	166	227
31–35	37	75	247	243
36–40	40	87	265	290
41–45	45	123	460	427
46–50	51	116	414	524
51–55	60	171	663	723
56–60	87	231	1,001	952
61–65	115	368	1,253	1,330
66–70	166	541	1,778	1,950
71–75	230	735	2,041	2,448
76–80	375	1,010	2,715	2,207
81–85	553	1,538	3,720	1,771
86–90	899	2,408	5,849	1,108
≥91	1,436	3,475	8,645	482

**Patients without heart failure regardless of kidney function**					**Patients with heart failure regardless of kidney function**				
Age (years)	NT-proBNP			N	Age (years)	NT-proBNP			N
	Q1 (ng/L)	Median (ng/L)	Q3 (ng/L)			Q1 (ng/L)	Median (ng/L)	Q3 (ng/L)	
≤25	27	56	164	261	≤25	111	395	3,214	19
26–30	28	50	140	203	26–30	140	1,479	4,956	24

31–35	35	69	209	226	31–35	310	767	3,580	17
36–40	38	72	206	256	36–40	108	389	1,557	34
41–45	36	88	262	354	41–45	311	902	3,238	73
46–50	45	91	233	437	46–50	327	880	2,910	87
51–55	50	112	298	545	51–55	281	1,046	3,345	178
56–60	67	143	498	676	56–60	378	1,107	3,613	276
61–65	82	183	590	908	61–65	514	1,371	3,831	422
66–70	115	281	817	1,202	66–70	600	1,547	4,229	748
71–75	149	347	1,059	1,355	71–75	659	1,514	3,750	1,093
76–80	207	505	1,220	1,061	76–80	736	1,850	4,402	1,146
81–85	301	756	1,825	683	81–85	952	2,287	4,719	1,088
86–90	398	953	2,541	326	86–90	1,375	3,255	7,600	782
≥91	667	1,600	3,785	107	≥91	1,854	4,054	10,048	375
**Patients without heart failure with creatinine-based eGFR ≥60**					**Patients with heart failure diagnosis with creatinine-based eGFR ≥60**				
Age (years)	NT-proBNP			N	Age (years)	NT-proBNP			N
	Q1 (ng/L)	Median (ng/L)	Q3 (ng/L)			Q1 (ng/L)	Median (ng/L)	Q3 (ng/L)	
≤25	27	56	164	261	≤25	111	320	2,831	3
26–30	28	50	140	203	26–30	101	753	4,355	2
31–35	35	69	209	226	31–35	269	642	2,675	1
36–40	38	72	206	354	36–40	103	242	1,222	5
41–45	36	88	262	437	41–45	290	650	2,675	9
46–50	45	91	233	545	46–50	302	790	2,194	17
51–55	50	112	298	676	51–55	264	789	2,066	38
56–60	67	143	498	908	56–60	340	944	2,463	51
61–65	82	183	590	1,202	61–65	500	1,233	2,778	105
66–70	115	281	817	1,355	66–70	473	1,140	2,682	274
71–75	149	347	1,059	1,061	71–75	460	1,312	2,896	496

76–80	207	505	1,220	683	76–80	621	1,345	2,980	692
81–85	301	756	1,825	326	81–85	588	1,511	3,335	779
86–90	398	953	2,541	107	86–90	1,140	2,085	4,475	643
≥91	667	1,600	3,785	437	≥91	979	2,570	5,910	341
**Males regardless of heart failure or kidney function**					**Females regardless of heart failure or kidney function**				
Age (years)	NT-proBNP			N	Age (years)	NT-proBNP			N
	Q1 (ng/L)	Median (ng/L)	Q3 (ng/L)			Q1 (ng/L)	Median (ng/L)	Q3 (ng/L)	
≤25	20	48	181	145	≤25	39	76	202	135
26–30	20	48	191	100	26–30	39	74	163	127
31–35	23	72	253	96	31–35	45	76	223	147
36–40	29	64	242	134	36–40	48	98	287	156
41–45	39	164	610	232	41–45	50	100	248	195
46–50	47	139	490	283	46–50	58	101	275	241
51–55	59	170	812	410	51–55	61	174	545	313
56–60	87	257	1,068	590	56–60	87	210	892	362
61–65	129	419	1,337	794	61–65	97	286	1,112	536
66–70	176	608	1,819	1,141	66–70	158	436	1,744	809
71–75	240	811	1,963	1,380	71–75	216	643	2,130	1,068
76–80	390	1,050	2,684	1,199	76–80	362	908	2,732	1,008
81–85	585	1,634	3,456	875	81–85	531	1,407	3,850	896
86–90	973	2,505	5,741	490	86–90	867	2,343	6,146	618
≥91	1,660	4,200	11,431	190	≥91	1,342	3,143	7,110	292
**Males without heart failure regardless of kidney function**					**Females without heart failure regardless of kidney function**				
Age (years)	NT-proBNP			N	Age (years)	NT-proBNP			N
	Q1 (ng/L)	Median (ng/L)	Q3 (ng/L)			Q1 (ng/L)	Median (ng/L)	Q3 (ng/L)	

≤25	20	43	159	133	≤25	37	73	188	128
26–30	15	40	148	90	26–30	38	58	132	113
31–35	21	60	178	86	31–35	44	72	212	140
36–40	28	55	185	117	36–40	46	92	230	139
41–45	28	85	365	178	41–45	46	90	204	176
46–50	40	90	215	217	46–50	55	93	234	220
51–55	45	103	279	294	51–55	57	128	321	251
56–60	65	143	538	400	56–60	70	142	464	276
61–65	84	207	603	509	61–65	82	161	563	399
66–70	111	301	826	671	66–70	122	243	786	531
71–75	155	347	999	723	71–75	144	343	1,120	632
76–80	205	505	1,179	543	76–80	213	507	1,268	518
81–85	306	807	1,934	339	81–85	295	719	1,736	344
86–90	470	1,093	2,848	144	86–90	363	878	2,253	182
≥91	710	1,635	5,686	32	≥91	575	1,566	3,720	75
**Males with heart failure regardless of kidney function**					**Females with heart failure regardless of kidney function**				
Age (years)	NT-proBNP			N	Age (years)	NT-proBNP			N
	Q1 (ng/L)	Median (ng/L)	Q3 (ng/L)			Q1 (ng/L)	Median (ng/L)	Q3 (ng/L)	
≤25	104	606	3,079	12	≤25	178	395	3,113	7
26–30	96	1,617	3,938	10	26–30	169	1,479	7,393	14
31–35	351	722	3,128	10	31–35	281	767	3,124	7
36–40	179	512	1,613	17	36–40	103	163	1,222	17
41–45	387	1443	3,744	54	41–45	194	637	1,222	19
46–50	323	824	2,950	66	46–50	415	1,210	2,550	21
51–55	348	1,092	3,273	116	51–55	281	954	4,044	62
56–60	371	1,107	3,461	190	56–60	403	1 155	4,046	86
61–65	620	1,390	3,804	285	61–65	358	1 318	3,835	137

66–70	618	1,544	3,942	470	66–70	538	1,553	4,457	278
71–75	714	1,503	3,438	657	71–75	586	1,570	4,095	436
76–80	769	1,918	4,170	656	76–80	701	1,784	4,478	490
81–85	1,057	2,324	4,728	536	81–85	843	2,185	4,582	552
86–90	1,422	3,326	6,790	346	86–90	1,360	3,230	7,927	436
≥91	2,255	4,785	12,300	158	≥91	1,750	3,710	8,700	217

Supplemental Data Table S2. Adjusted and non-adjusted correlations for NT-proBNP.

**NT-proBNP**	**r-value**	***P*-value**	**Adjusted**	**N**
**LVEF[1] – All**	-0.404	<0.0001	no	14,962
**Male**	-0.450	<0.0001	no	8,059
**Female**	-0.358	<0.0001	no	6,903
**LVEF[1] – All**	-0.315	<0.0001	Age ≥60 years	14,962
**Male**	-0.309	<0.0001	Age ≥60 years	8,059
**Female**	-0.346	<0.0001	Age ≥60 years	6,903
**LVEF[1] – All**	-0.284	<0.0001	Creatinine >90/105 micromol/L	14,466
**Male**	-0.279	<0.0001	Creatinine >90/105 micromol/L	7,834
**Female**	-0.319	<0.0001	Creatinine >90/105 micromol/L	6,632
**LVEF[1] – All**	-0.294	<0.0001	Creatinine-based eGFR <60 mL/min/1.73m^2 BSA	14,466
**Male**	-0.287	<0.0001	Creatinine-based eGFR <60 mL/min/1.73m^2 BSA	7,834
**Female**	-0.323	<0.0001	Creatinine-based eGFR <60 mL/min/1.73m^2 BSA	6,632
**LVEF[1] – All**	-0.222	<0.0001	Cystatin C >1.10/1.20/1.30/1.40 mg/L	3,059
**Male**	-0.235	<0.0001	Cystatin C >1.10/1.20/1.30/1.40 mg/L	1,836
**Female**	-0.237	<0.0001	Cystatin C >1.10/1.20/1.30/1.40 mg/L	1,223
**LVEF[1] – All**	-0.225	<0.0001	Cystatin C-based eGFR <60 mL/min/1.73m^2 BSA	3,059
**Male**	-0.233	<0.0001	Cystatin C-based eGFR <60 mL/min/1.73m^2 BSA	1,836
**Female**	-0.247	<0.0001	Cystatin C-based eGFR <60 mL/min/1.73m^2 BSA	1,223
**LVEF[1] – All**	-0.204	<0.0001	Age, Creatinine and eGFR, Cystatin C and eGFR	3,053
**Male**	-0.216	<0.0001	Age, Creatinine and eGFR, Cystatin C and eGFR	1,835

**Female**	-0.221	<0.0001	Age, Creatinine and eGFR, Cystatin C and eGFR	1,218
**Age – All**	0.492	<0.0001	no	14,962
**Male**	0.463	<0.0001	no	8,059
**Female**	0.526	<0.0001	no	6,903
**Creatinine – All**	0.348	<0.0001	no	14,466
**Male**	0.354	<0.0001	no	7,834
**Female**	0.388	<0.0001	no	6,632
**Creatinine-based eGFR – All**	-0.515	<0.0001	no	14,466
**Male**	-0.498	<0.0001	no	7,834
**Female**	-0.536	<0.0001	no	6,632
**Cystatin C – All**	0.534	<0.0001	no	3,059
**Male**	0.518	<0.0001	no	1,836
**Female**	0.557	<0.0001	no	1,223
**Cystatin C-based eGFR – All**	-0.545	<0.0001	no	3,059
**Male**	-0.530	<0.0001	no	1,836
**Female**	-0.568	<0.0001	no	1,223

[1]Left ventricular ejection fraction

Supplemental Data Table S3. Time to heart failure diagnosis based on NT-proBNP and left ventricular ejection fraction.

Heart failure diagnosis	Median (days)	95% CI (days)	N
**NT-proBNP <125 ng/L – All**	596	448–835	143
**Male**	469	260–818	91
**Female**	819	469–1,317	52
**NT-proBNP <300 ng/L – All**	639	469–762	419
**Male**	495	350–695	246
**Female**	777	596–946	173
**NT-proBNP ≥125 ng/L – All**	62	53–76	3,168
**Male**	60	51–76	1,749

**Female**	63	49–92	1,419
**NT-proBNP ≥300 ng/L – All**	49	41–58	2,892
**Male**	49	39–60	1,594
**Female**	49	29–64	1,298
**LVEF[1] ≤40% – All**	44	33–58	610
**Male**	56	39–72	434
**Female**	21	8–49	176
**LVEF[1] 41–49% – All**	131	85–189	312
**Male**	107	63–189	198
**Female**	156	73–333	114
**LVEF[1] ≥50% – All**	596	531–639	1,399
**Male**	597	502–672	684
**Female**	594	513–666	715

[1]Left ventricular ejection fraction
